# Stochastic model of the transmission dynamics of COVID-19 pandemic

**DOI:** 10.1186/s13662-021-03597-1

**Published:** 2021-10-18

**Authors:** Aychew Wondyfraw Tesfaye, Tesfaye Sama Satana

**Affiliations:** Department of Mathematics, College of Natural and Computational Sciences, Haramaya University, Dire Dawa, Ethiopia

**Keywords:** COVID-19 disease, Stochastic model, Numerical simulation

## Abstract

In this paper, we formulate an *SVITR* deterministic model and extend it to a stochastic model by introducing intensity of stochastic factors and Brownian motion. Our basic qualitative analysis of both models includes the positivity of the solution, invariant region, disease-free equilibrium point, basic reproduction number, local and global stability of disease-free equilibrium point, endemic equilibrium point, and sensitivity. We obtain the stochastic reproduction number and local stability by using twice differentiable Itô’s formula. We prove the global stability of the disease-free equilibrium point by using a Lyapunov function. We determine the sensitivity of the effect of each parameter on basic reproduction number of the model by using a normalized sensitivity index formula. On the other hand, we demonstrate numerical simulation results of deterministic and stochastic models of COVID-19 by using Maple 18 and MATLAB software. Our simulation results indicate that reducing the contact between infected and susceptible individuals and improvement of treatment play a vital role in COVID-19 pandemic control.

## Introduction

Mathematical modeling is useful in understanding and analyzing the behavior of infectious disease transmission dynamics in humans and animals. Since the first discovery and identification of coronavirus in 1965, three major outbreaks occurred, which were caused by emerging and highly pathogenic coronavirus. In 2003, in Mainland China, the outbreak of Sever Acute Respiratory Syndrome Coronavirus (SARS-CoV) occurred [5, 12]; in 2012 the outbreak of Middle East Respiratory Syndrome Coronavirus (MERS-CoV) in Saudi Arabia occurred [16], and the virus MERS-CoV in South Korea happened [3, 10]. Currently, Corona Virus Disease 2019 (COVID-19) is an infectious disease caused by Sever Acute Respiratory Syndrome Coronavirus 2 (SARS-CoV-2). The infection was noticed in Wuhan, China, where by the first case of infection was identified in December 2019 [2, 11, 13]. The outbreak was declared a public health emergency of international concern on 30 January 2020 by World Health Organization (WHO).

The World Health Organization renamed COVID-19 as strain Severe Acute Respiratory Syndrome Corona Virus 2 (SARS-CoV-2) on 11 February 2020 [4, 20]. As of 17 March 2021, more than 120 million cases of COVID-19 infections, with more than 2 million deaths, had been reported globally [20]. According to Center for Diseases Control (CDC) [1], the major symptoms of COVID-19 are fever, cough or sneeze, and breath shortness, which emerges 14 days after the infection occurred. Public health [18] and WHO [20] claimed that the virus that causes COVID-19 spreads mainly when an infected person is in close contact with another person. Small droplets and aerosols containing the virus can spread from an infected person’s nose and mouth when they breathe, cough, sneeze, sing, or speak [21]. The virus may also spread via contaminated surfaces. Based on the spreading nature of COVID-19, WHO declared it as a global pandemic on 11 March 2020 [20].

It is known that there is no known medicine to combat the novel coronavirus pandemic yet [14]. WHO made certain standardized recommendations to prevent the spread of COVID-19. They include frequent washing of hands with soap and alcohol-base sanitizer, using face mask, and avoiding close contact with anyone that has a fever and cough [21]. A COVID-19 vaccine is intended to provide acquired immunity against (SARS-CoV-2). In phase III trials, several COVID-19 vaccines have demonstrated efficacy as high as 95% in preventing symptomatic COVID-19 infections. So far, more than 20 million doses of the AstraZeneca vaccine have been administered in Europe, and more than 27 million doses of the Covishield vaccine (AstraZeneca vaccine by Serum Institute of India) have been administered in India. As of 17 April 2021, 890.31 million doses of COVID-19 vaccine have been administered worldwide by official reports from national health agencies [9].

Mathematical models are important tools to gain a big understanding of the ongoing trends for COVID-19. They are also useful for obtaining a basic reproduction number, determining sensitivities to change in parameter values, estimating key parameters from the data that contribute to identifying trends, making general forecasts, and estimating uncertainties [6]. Epidemiological models play a fundamental role in the study of the dynamics of COVID-19. With regard to the studies carried out so far, a few mathematical modeling studies have been done about transmition of the pandemic. For instance, Wu et al. [22] introduced a susceptible-exposed-infectious-recovered (SEIR) model to describe the transmission dynamics and forecasted the national and global spread of the disease. The model is considered simple and does not incorporate the relapse form recovered class to susceptible class, which is very unrealistic. The author did not introduce any treatment and vaccination classes. A similar study was done by Read et al. [15], but with no vaccination and treatment classes. As COVID-19 is a chronic disease, treatment should be considered to make the work more realistic, which is missing in [15]. Tang et al. [17] proposed a deterministic compartmental model incorporating the clinical progression of the disease, the individual epidemiological status, and the intervention measures. They found that the control reproductive number could be as high as 6.47, and those intervention strategies such as intensive contact tracing followed by quarantine and isolation can effectively reduce the control reproduction number and the transmission risk. Imai et al. [8] conducted computational modeling of potential epidemic trajectories to estimate the size of the disease outbreak in Wuhan, with a focus on the human-to-human transmission. Their findings showed that control measures need to block well over 60% of transmission to be effective in containing the outbreak.

Recently, Iboi et al. [7] developed a mathematical model to determine whether or not a hypothetical imperfect vaccine can lead to the elimination of COVID-19 in the United States. Their study indicated that such elimination is feasible, using the hypothetical vaccine with assumed efficacy of 80%, if the vaccine coverage is adequate enough to achieve herd immunity. In particular, the vaccine coverage needed to achieve herd immunity in US is 90%, whereas the computed herd thresholds for the states of New York and the state of Florida are 84% and 85%, respectively. Therefore from the above discussion we can conclude that human-to-human contact is the potential cause of outbreaks of COVID-19. Thus isolation of an infected human can reduce the risk of future spread of COVID-19. Thus, to overcome those limitations, we conducted the current study to develop a stochastic SVITR mathematical model for the transmission dynamics of COVID-19 pandemic by introducing treated and vaccinated classes.

The paper is organized as follows. In Sect. 2, we formulate and describe the mathematical model. In Sect. 3, we qualitatively analyze the model by examining the equilibrium points. Numerical simulations of the model by estimating the parameters are given in Sect. 4, where the sensitivity of the basic reproduction number on the model parameters is also discussed. Last but not least, conclusions and recommendations of the study are given in Sect. 5.

## Model description

The model we have already formulated consists of five compartments: susceptible $S(t)$, vaccinated $V (t)$, infected $I(t)$, treated $T(t)$, and recovered $R(t)$ individuals for all times $t > 0$. The susceptible are those individuals that are not infected by COVID-19 but can infect in future. Infected individuals include individuals that are couched by COVID-19 and are able to transmit the disease to a susceptible one. Treated individuals are those who can be treated from the disease and cannot transmit to other individuals. After treatment class, some individuals are recovered from the disease. The susceptible populations are increased by recruitment rate *φ*, either by birth or immigration. Those individuals can be vaccinated at rate *θ* and become infected by COVID-19 with contact rate *α*. The infected individuals are decreased by disease causing death rate *τ*. The infected individuals can get treatment at rate *δ*, and those individuals that are in treatment can recover from the disease at rate *ρ*. The vaccinated and recovered individuals can lose their temporary immunity at rates $\sigma _{2}$ and $\sigma _{1}$, respectively, becoming susceptible again. We have natural causing death rate *μ* for the whole population.

Our model is governed by the following assumptions: All parameters are nonnegative, the total population size is constant, vaccination is introduced to the susceptible individuals, susceptible individuals are recruited by birth or immigration, the treated individuals cannot transmit COVID-19 disease to the susceptible population, and by losing temporary immunity the recovered individuals become susceptible again. The compartments and parameters of the model are described in Table 1.

**Table 1:** Description of state variables and parameters

Notations	Description
*S*(*t*)	Number of susceptible individuals at time *t*
*V*(*t*)	Number of vaccinated individuals at time *t*
*I*(*t*)	Number of infected individuals at time *t*
*T*(*t*)	Number of treated individuals at time *t*
*R*(*t*)	Number of recovered individuals at time *t*
*φ*	Recruitment rate of susceptible individuals due to migration or birth
*α*	The rate at which susceptible individuals tend to infected individuals
*δ*	Treatment rate
*μ*	Natural causing death rate of all individuals
*θ*	Vaccination rate
*τ*	Rate of death due to COVID-19
*ρ*	Recovery rate of treated individuals
$\sigma _{1}$	Lose rate of immunity by recovered individuals
$\sigma _{2}$	Lose rate of immunity by vaccinated individuals
*ω*	COVID-19 causing death rate associated with the individuals that are on treatment
*B*(*t*)	Standard Brownian motions
$\beta _{i}$	Intensity of white noise

**Figure 1:**
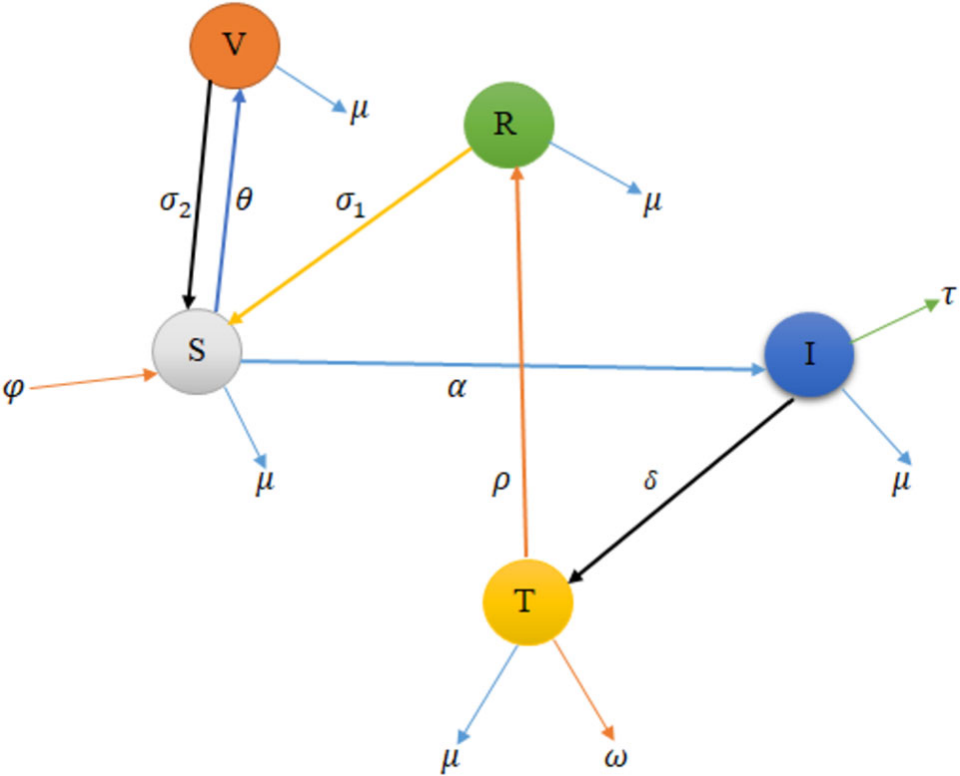
Schematic diagram of COVID-19 model

The model is shown diagrammatically in Fig. 1.

The deterministic *SVITR* model is as follows:

2.1 $$ \textstyle\begin{cases} \frac{dS(t)}{dt} =\varphi + \sigma _{1} R ( t ) + \sigma _{2} V ( t ) - ( \alpha I ( t ) +\theta +\mu ) S ( t ),\\ \frac{dV}{dt} = \theta S ( t ) - ( \mu + \sigma _{2} ) V ( t ), \\ \frac{dI(t)}{dt} =\alpha S ( t ) I ( t ) - ( \mu +\delta +\tau ) I ( t ), \\ \frac{dT(t)}{dt} =\delta I ( t ) - ( \mu +\rho +\omega ) T ( t ), \\ \frac{dR(t)}{dt} =\rho T ( t ) - ( \mu + \sigma _{1} ) R ( t ), \end{cases} $$

with initial conditions $S ( 0 ) = S_{0}$, $V ( 0 ) = V_{0}$, $I ( 0 ) = I_{0}$, $T ( 0 ) = T_{0}$, $R(0) = R_{0} $.

Incorporating the intensity of stochastic environmental factors $\beta _{I}$ and Brownian motion $B_{I}(t)$ into the *SVITR* deterministic model Eq. (2.1), we get the corresponding stochastic model of COVID-19:

2.2 $$ \textstyle\begin{cases} dS ( t ) = ( \varphi + \sigma _{1} R ( t ) + \sigma _{2} V ( t ) - ( \alpha I ( t ) +\theta +\mu ) S ( t ) ) \,dt+ \beta _{1} S ( t ) \,d B_{1} ( t ),\\ dV ( t ) = ( \theta S ( t ) - ( \mu + \sigma _{2} ) V ( t ) ) \,dt+ \beta _{2} V ( t ) \,d B_{2} ( t ), \\ dI ( t ) = ( \alpha S ( t ) I ( t ) - ( \mu +\delta +\tau ) I ( t ) ) \,dt+ \beta _{3} I ( t ) \,d B_{3} ( t ), \\ dT ( t ) = ( \delta I ( t ) - ( \mu +\rho +\omega ) T ( t ) ) \,dt+ \beta _{4} T ( t ) \,d B_{4} ( t ), \\ dR ( t ) = ( \rho T ( t ) - ( \mu + \sigma _{1} ) R ( t ) ) \,dt+ \beta _{5} R ( t ) \,d B_{5} ( t ), \end{cases} $$

where $\beta _{1}, \beta _{2}, \beta _{3}, \beta _{4}, \beta _{5} \geq 0$ (intensities of Brownian motions), and $B_{1}$, $B_{2}$, *B*, $B_{4}$, $B_{5}$ are independent Brownian motions.

## Qualitative analysis

In this section, we discuss the qualitative behavior of the model.

### Positivity of the solution

#### Theorem 3.1

*Let* $S_{0} \geq 0$, $V_{0} \geq 0$, $I_{0} \geq 0$, $T_{0} \geq 0$, $R_{0} \geq 0$. *Then* $\{S ( t ), V ( t ), I ( t ), T ( t ),R(t) \}$ *are positive for all times* *t*.

#### Proof

First, let us compute $\frac{dS}{dt} $ from model Eq. (2.1):

3.1 $$\begin{aligned}& \frac{dS(t)}{dt} =\varphi + \sigma _{1} R ( t ) + \sigma _{2} V ( t ) - \bigl( \alpha I ( t ) +\theta +\mu \bigr) S(t) \\& \quad \Rightarrow\quad \frac{dS(t)}{dt} \geq - \bigl( \alpha I ( t ) +\theta +\mu \bigr) S(t) \\& \quad \Rightarrow\quad \frac{dS(t)}{ ( \alpha I ( t ) +\theta +\mu ) S(t)} \geq -dt. \end{aligned}$$

Then applying the initial conditions and solving by using separation of variables, we get

3.2 $$\begin{aligned}& S ( t ) \geq e^{c} e^{ ( \alpha I ( t ) +\theta +\mu ) t}, \\& S ( t ) \geq S ( 0 ) e^{ ( \alpha I ( t ) +\theta +\mu ) t}. \end{aligned}$$

Next, from the second equation of (2.1) we get

3.3 $$\begin{aligned}& \frac{dV(t)}{dt} =\theta S ( t ) - ( \mu + \sigma _{2} ) V ( t ) \\& \quad \Rightarrow\quad \frac{dV(t)}{dt} \geq - ( \mu + \sigma _{2} ) V ( t ) \end{aligned}$$

3.4 $$\begin{aligned}& \quad \Rightarrow\quad \frac{dV(t)}{ ( \mu + \sigma _{2} ) V(t)} \geq -dt. \end{aligned}$$

Therefore $V(t) \geq V ( 0 ) e^{ ( \mu + \sigma _{2} ) t}$.

Finally, in a similar way, from the third, fourth, and fifth equations of model (2.1) we get

$$\begin{aligned}& I ( t ) \geq I ( 0 ) e^{ ( \mu +\delta +\tau ) t}, \\& T ( t ) \geq T ( 0 ) e^{ ( \mu +\rho +\omega ) t}, \\& R ( t ) \geq R ( 0 ) e^{ ( \mu + \sigma _{1} ) t}. \end{aligned}$$

Therefore our model is positive for all $t \geq 0$. □

### Invariant region

To get the region at which the model is bounded, let us take the total population $N(t)$ for all *t*,

3.5 $$ N ( t ) =S ( t ) +V ( t ) +I ( t ) +T ( t ) +R ( t ). $$

By differentiating Eq. (3.5) with respect to *t* we have

3.6 $$\begin{aligned}& \frac{dN(t)}{dt} = \frac{dS(t)}{dt} + \frac{dV(t)}{dt} + \frac{dI(t)}{dt} + \frac{dT(t)}{dt} + \frac{dR(t)}{dt}, \\ \end{aligned}$$

3.7 $$\begin{aligned}& \frac{dN(t)}{dt} =\varphi -\mu \bigl( S ( t ) +V ( t ) +I ( t ) +T ( t ) +R ( t ) \bigr) -\tau I ( t ) -\omega T ( t ). \end{aligned}$$

In the absence of death due to COVID-19 ($\tau =0$ and $\omega =0$), Eq. (3.7) becomes

3.8 $$ \frac{dN(t)}{dt} =\varphi -\mu N ( t ). $$

Then solving Eq. (3.8) and applying the initial conditions, we get

3.9 $$ N ( t ) \leq \frac{\varphi }{\mu } - \frac{N(0)}{\mu } e^{- \mu t}. $$

As $t\longrightarrow \infty $ in Eq. (3.9), we obtain

3.10 $$\begin{aligned}& \lim_{t\longrightarrow \infty } N ( t ) = \frac{\varphi }{\mu }, \\& 0 \leq N ( t ) \leq \frac{\varphi }{\mu }. \end{aligned}$$

Thus our model is positively invariant in the region

$$ \Omega = \biggl\{ ( S, V, I, T, R ) \in \mathbb{R}_{+}^{5}: 0 \leq N ( t ) \leq \frac{\varphi }{\mu } \biggr\} . $$

### Disease-free equilibrium point

In this section, we start with no COVID-19 present in the population of human, so that to obtain COVID-19-free equilibrium point, let us take $I = T = R = 0$. Now we have left with susceptible and vaccinated individuals from model Eq. (2.1):

3.11 $$ \textstyle\begin{cases} \frac{dS(t)}{dt} =\varphi + \sigma _{1} R ( t ) + \sigma _{2} V ( t ) - ( \alpha I ( t ) +\theta +\mu ) S ( t ),\\ \frac{dV(t)}{dt} =\theta S ( t ) - ( \mu + \sigma _{2} ) V ( t ). \end{cases} $$

Finally, we get the following points

$$ S_{0} = \frac{\varphi (\mu + \sigma _{2} )}{\mu ( \mu + \sigma _{2} +\theta )},\qquad V_{0} = \frac{\varphi \theta }{\mu ( \mu + \sigma _{2} +\theta )} $$

since $I =0$, $T =0$, and $R = 0$.

Thus the disease-free equilibrium lies at the point $E_{0} = ( \frac{\varphi (\mu + \sigma _{2} )}{\mu ( \mu + \sigma _{2} +\theta )}, \frac{\varphi \theta }{\mu ( \mu + \sigma _{2} +\theta )}, 0, 0, 0 ) $.

### Basic reproduction number

In this section, we obtain both deterministic and stochastic reproduction numbers.

#### Basic reproduction number in the deterministic model

To get basic reproduction number, consider the newly infectious class of model Eq. (2.1):

$$ \frac{dI(t)}{dt} =\alpha S ( t ) I(t)- ( \mu +\delta +\tau ) I ( t ). $$

Let *f* be the rate of appearance of new infection, and let *v* be the rate of transfer of new infection into and out of the compartment. Then

$$\begin{aligned}& f=\alpha S ( t ) I ( t ), \\& v= ( \mu +\delta +\tau ) I ( t ). \end{aligned}$$

Then we obtain the Jacobian matrices of *f* and *v* (represented by *F* and *V*) with respect to *I*:

3.12 $$\begin{aligned}& F= \frac{\partial f}{\partial I} =\alpha S ( t ), \end{aligned}$$

3.13 $$\begin{aligned}& V= \frac{\partial v}{\partial I} = ( \mu +\delta +\tau ). \end{aligned}$$

Then *F* and *V* at $E_{0} = ( \frac{\varphi (\mu + \sigma _{2} )}{\mu (\mu + \sigma _{2} +\theta )}, \frac{\varphi \theta }{\mu (\mu + \sigma _{2} +\theta )}, 0, 0, 0 )$ become

$$\begin{aligned}& F= \frac{\alpha \varphi (\mu + \sigma _{2} )}{\mu (\mu + \sigma _{2} +\theta )}, \\& v= ( \mu +\delta +\tau ), \qquad v^{-1} = \frac{1}{ ( \mu +\delta +\tau )}. \end{aligned}$$

Then spectral radius ($F V^{-1}$) of our model becomes

$$ F V^{-1} = \frac{\alpha \varphi (\mu + \sigma _{2} )}{\mu (\mu + \sigma _{2} +\theta ) ( \mu +\delta +\tau )}. $$

Therefore our basic reproduction number in the deterministic model is

3.14 $$ R_{0}^{D} = \frac{\alpha \varphi (\mu + \sigma _{2} )}{\mu (\mu + \sigma _{2} +\theta ) ( \mu +\delta +\tau )}. $$

#### Basic reproduction number in the stochastic model

To obtain the stochastic basic reproduction number, we consider the infected class of stochastic model Eq. (2.2):

3.15 $$ dI ( t ) = \bigl[ \alpha S ( t ) - ( \mu +\delta +\tau ) I(t) \bigr] \,dt+ \beta _{3} I ( t ) \,d B_{3} ( t ). $$

Now by using Itô’s formula we derive our basic reproduction number in the stochastic approach. Let as consider the Taylor series expression of $g ( t, I ( t ) ) = \ln ( I ( t ) )$:

3.16 $$\begin{aligned} dg \bigl( t, I ( t ) \bigr) &= \frac{\partial g}{\partial t} \,dt+ \frac{\partial g}{\partial I ( t )} \,dI ( t ) + \frac{1}{2} \frac{\partial ^{2} g}{\partial I^{2}} ( dI )^{2} \\ &\quad {}+ \frac{\partial ^{2} g}{\partial t\,\partial I} \,dt \,dI ( t ) + \frac{1}{2} \frac{\partial ^{2} g}{\partial t^{2}} ( dt )^{2}, \end{aligned}$$

where $\frac{\partial g}{\partial t} =0$, $\frac{\partial g}{\partial I(t)} = \frac{1}{I(t)}$, $\frac{\partial ^{2} g}{\partial I^{2}} = \frac{1}{I^{2} (t)}$, $\frac{\partial ^{2} g}{\partial t\,\partial I} =0$, $\frac{\partial ^{2} g}{\partial t^{2}} =0$,

$$\begin{aligned} dg \bigl( t, I ( t ) \bigr) &= \frac{1}{I ( t )} \,dI(t)- \frac{1}{2 I^{2} ( t )} \,d I^{2} ( t ) \\ &= \frac{1}{I ( t )} \bigl[ \bigl( \alpha S ( t ) - ( \mu +\delta +\tau ) I(t) \bigr) \,dt+ \beta _{3} I ( t ) \,d B_{3} ( t ) \bigr] \\ &\quad {}- \frac{1}{2 I^{2} ( t )} \bigl[ \bigl( \alpha S ( t ) - ( \mu +\delta +\tau ) I ( t ) \bigr) \,dt+ \beta _{3} I ( t ) \,d B_{3} ( t ) \bigr]^{2}. \end{aligned}$$

Let $e=\alpha S ( t ) I ( t ) - ( \mu +\delta +\tau ) I ( t )$ and $f= \beta _{3} I ( t ) $. Then

$$\begin{aligned} dg \bigl( t, I ( t ) \bigr) &= \bigl[ \alpha S ( t ) - ( \mu +\delta +\tau ) \bigr] \,dt+ \beta _{3} \,d B_{3} ( t ) \\ &\quad {}- \frac{1}{2 I^{2} ( t )} \bigl[ e \,dt+f\,d B_{3} ( t ) \bigr]^{2} \\ &= \bigl[ \alpha S ( t ) - ( \mu +\delta +\tau ) \bigr] \,dt+ \beta _{3} \,d B_{3} ( t ) \\ &\quad {}- \frac{1}{2 I^{2} ( t )} \bigl[ e^{2} d^{2} t+2ef\,dt \,d B_{3} ( t ) + f^{2}\, d^{2} B_{3} ( t ) \bigr] \\ &= \bigl[ \alpha S ( t ) - ( \mu +\delta +\tau ) \bigr] \,dt+ \beta _{3} \,d B_{3} ( t ) - \frac{1}{2 I^{2} ( t )} \bigl[ f^{2} \,d^{2} B_{3} ( t ) \bigr]. \end{aligned}$$

Then by applying the chain rule we get

$$\begin{aligned} dg\bigl(t, I ( t ) \bigr)&= \bigl[ \alpha S ( t ) - ( \mu +\delta +\tau ) \bigr] \,dt+ \beta _{3} \,d B_{3} ( t ) - \frac{1}{2 I^{2} ( t )} \bigl[ \beta _{3}^{2} I^{2} (t) \,d^{2} B_{3} ( t ) \bigr] \\ &= \bigl[ \alpha S ( t ) - ( \mu +\delta +\tau ) \bigr] \,dt+ \beta _{3} \,d B_{3} ( t ) - \frac{1}{2} \bigl[ \beta _{3}^{2} \,dt \bigr] \\ &= \biggl[ \alpha S ( t ) - \frac{1}{2} \beta _{3}^{2} - ( \mu +\delta +\tau ) \biggr] \,dt+ \beta _{3} \,d B_{3} ( t ). \end{aligned}$$

Let *f* be the rate of introduction of new infection, and let *v* be the rate of transfer of new infection. Then *f* and *v* at disease-free equilibrium point become

$$\begin{aligned}& f= \biggl[ \frac{\alpha \varphi (\mu + \sigma _{2} )}{\mu (\mu + \sigma _{2} +\theta )} - \frac{1}{2} \beta _{3}^{2} \biggr], \\& v= ( \mu +\delta +\tau ), \\& v^{-1} = \frac{1}{ ( \mu +\delta +\tau )}. \end{aligned}$$

Then the product of *f* and $v^{-1}$ equals

3.17 $$ fv^{-1} = \frac{\alpha \varphi (\mu + \sigma _{2} )}{\mu (\mu + \sigma _{2} +\theta ) ( \mu +\delta +\tau )} - \frac{\beta _{3}^{2}}{2 ( \mu +\delta +\tau )}, $$

where $R_{0}^{D} = \frac{\alpha \varphi (\mu + \sigma _{2} )}{\mu (\mu + \sigma _{2} +\theta ) ( \mu +\delta +\tau )} $.

Therefore our basic reproduction number in the stochastic model is

3.18 $$ R_{0}^{S} = R_{0}^{D} - \frac{\beta _{3}^{2}}{2 ( \mu +\delta +\tau )}. $$

### Local stability of disease-free equilibrium point

#### Local stability of disease-free equilibrium point for deterministic

##### Theorem 3.2

*The disease*-*free equilibrium point is locally asymptotically stable if and only if* $R_{0} <1$ *and unstable otherwise*.

##### Proof

To prove this theorem, we use the Jacobian matrix of model Eq. (2.1),

3.19 $$ J = \begin{pmatrix} -(\mu +\theta ) & \sigma _{2} & -\alpha {S}({t}) & 0 & \sigma _{1}\\ \theta & -(\mu + \sigma _{2} ) & 0 & 0 & 0\\ 0 & 0 & \alpha {S}({t})-(\mu +\delta +\tau ) & 0 & 0\\ 0 & 0 & \delta & -(\mu +\rho +\omega ) & 0\\ 0 & 0 & 0 & \rho & -(\mu + \sigma _{2} ) \end{pmatrix}. $$

Then solving Eq. (3.19) at the disease-free equilibrium point $E_{0} = ( \frac{\varphi (\mu + \sigma _{2} )}{\mu (\mu + \sigma _{2} +\theta )}, \frac{\varphi \theta }{\mu (\mu + \sigma _{2} +\theta )}, 0, 0, 0 )$, we obtain

3.20 $$\begin{aligned}& J = \begin{pmatrix} -(\mu +\theta ) & \sigma _{2} & - \frac{\alpha \varphi (\mu + \sigma _{2} )}{\mu (\mu + \sigma _{2} +\theta )} & 0 & \sigma _{1}\\ \theta & -(\mu + \sigma _{2} ) & 0 & 0 & 0\\ 0 & 0 & \frac{\alpha \varphi (\mu + \sigma _{2} )}{\mu (\mu + \sigma _{2} +\theta )} -(\mu +\delta +\tau ) & 0 & 0\\ 0 & 0 & \delta & -(\mu +\rho +\omega ) & 0\\ 0 & 0 & 0 & \rho & -(\mu + \sigma _{2} ) \end{pmatrix}. \end{aligned}$$

The roots of Eq. (3.20) can be obtained from

3.21 $$\begin{aligned}& J = \begin{pmatrix} - ( \mu +\theta ) - \lambda & \sigma _{2} & - \frac{\alpha \varphi (\mu + \sigma _{2} )}{\mu (\mu + \sigma _{2} +\theta )} & 0 & \sigma _{1}\\ \theta & - ( \mu + \sigma _{2} ) - \lambda & 0 & 0 & 0\\ 0 & 0 & \frac{\alpha \varphi (\mu + \sigma _{2} )}{\mu (\mu + \sigma _{2} +\theta )} - ( \mu +\delta +\tau ) - \lambda & 0 & 0\\ 0 & 0 & \delta & - ( \mu +\rho +\omega ) - \lambda & 0\\ 0 & 0 & 0 & \rho & - ( \mu + \sigma _{2} ) - \lambda \end{pmatrix}. \end{aligned}$$

From (3.21) we obtain the characteristic equation

3.22 $$ \begin{aligned}[b] &\biggl[ - ( \mu +\theta ) - \lambda \bigl[ - ( \mu + \sigma _{2} ) - \lambda \bigr] \biggl[ \frac{\alpha \varphi ( \mu + \sigma _{2} )}{\mu ( \mu + \sigma _{2} +\theta )} - ( \mu +\delta +\tau ) - \lambda \biggr] \\ &\quad {}\times\bigl[ - ( \mu +\rho +\omega ) - \lambda \bigr] \bigl[ - ( \mu + \sigma _{2} ) - \lambda \bigr] \biggr] =0. \end{aligned} $$

Obviously, $\lambda _{1}$, $\lambda _{2}$, $\lambda _{3}$, and $\lambda _{4}$ are negative. For stability, all the real parts of the eigenvalues must be negative, which means

3.23 $$ \frac{\alpha \varphi ( \mu + \sigma _{2} )}{\mu ( \mu + \sigma _{2} +\theta )} - ( \mu +\delta +\tau ) < 0. $$

After rearranging Eq. (3.23), we get

3.24 $$ \frac{\alpha \varphi ( \mu + \sigma _{2} )}{\mu ( \mu + \sigma _{2} +\theta ) ( \mu +\delta +\tau )} < 1. $$

Now from Eq. (3.24) we observe that

$$ R_{0}^{D} = \frac{\alpha \varphi ( \mu + \sigma _{2} )}{\mu ( \mu + \sigma _{2} +\theta ) ( \mu +\delta +\tau )} < 1. $$

Therefore our disease-free equilibrium point is locally asymptotically stable if $R_{0}^{D} <1$. □

#### Local stability of disease-free equilibrium point in the stochastic model

##### Theorem 3.3

*If* $R_{0}^{S} <1$, *then for any initial values of* $( S_{0}, V_{0}, I_{0}, T_{0}, R_{0} ) \in R_{+}^{5}$, $I(t)$ *obeys* $\lim_{t\rightarrow \infty } \sup \frac{1}{t} \ln I ( t ) \leq ( \mu +\delta +\tau ) (R_{0}^{S} -1)<0$.

##### Proof

Let as take the Taylor series expansion of $F(t, I(t)) = \ln I(t)$. By Itô’s formula

3.25 $$\begin{aligned}& dF \bigl( t, I ( t ) \bigr) = \biggl[ \alpha S ( t ) - \frac{1}{2} \beta _{3}^{2} - ( \mu +\delta +\tau ) \biggr] \,dt+ \beta _{3} \,d B_{3} ( t ), \\& d \ln I(t) = \biggl[ \alpha S ( t ) - \frac{1}{2} \beta _{3}^{2} - ( \mu +\delta +\tau ) \biggr] \,dt+ \beta _{3} \,d B_{3} ( t ). \end{aligned}$$

Now integrating both sides of Eq. (3.25), we get

3.26 $$ \ln I(t) - \ln I ( 0 ) = \int _{0}^{t} \biggl[ \alpha S ( t ) - \frac{1}{2} \beta _{3}^{2} - ( \mu +\delta +\tau ) \biggr] \,dt+ \int _{0}^{t} \beta _{3} \,d B_{3} ( t ). $$

Then solving Eq. (3.26) at disease-free equilibrium point, we have

3.27 $$\begin{aligned}& \ln I(t) = \ln I ( 0 ) + \int _{0}^{t} \biggl[ \frac{\alpha \varphi (\mu + \sigma _{2} )}{\mu (\mu + \sigma _{2} +\theta )} - \frac{1}{2} \beta _{3}^{2} - ( \mu +\delta +\tau ) \biggr] \,dt+ \int _{0}^{t} \beta _{3} \,d B_{3} ( t ) \\& \quad \Rightarrow \quad \ln I(t) \leq \ln I ( 0 ) + \biggl( \frac{\alpha \varphi (\mu + \sigma _{2} )}{\mu (\mu + \sigma _{2} +\theta )} - \frac{1}{2} \beta _{3}^{2} - ( \mu +\delta +\tau ) \biggr) t+G ( t ), \end{aligned}$$

where $G ( t ) = \int _{0}^{t} \beta _{3} \,d B_{3} ( t )$ is a martingale.

By using the strong law of martingales we have $\lim_{t\rightarrow \infty } \sup \frac{G(t)}{t} =0$ almost surely.

Then divide both sides of Eq. (3.27) by *t*:

3.28 $$ \Rightarrow \frac{\ln I(t)}{t} \leq \frac{\ln I ( 0 )}{t} + \biggl( \frac{\alpha \varphi ( \mu + \sigma _{2} )}{\mu ( \mu + \sigma _{2} +\theta )} - \frac{1}{2} \beta _{3}^{2} - ( \mu +\delta +\tau ) \biggr) + \frac{G ( t )}{t}. $$

Now taking $\lim_{t\rightarrow \infty } \sup $ of (3.28), we obtain

3.29 $$\begin{aligned}& \lim_{t\rightarrow \infty } \sup \frac{\ln I(t)}{t} \\& \quad \leq \lim _{t\rightarrow \infty } \sup \frac{\ln I ( 0 )}{t} + \biggl( \frac{\alpha \varphi ( \mu + \sigma _{2} )}{\mu ( \mu + \sigma _{2} +\theta )} - \frac{1}{2} \beta _{3}^{2} - ( \mu +\delta +\tau ) \biggr) + \lim_{t\rightarrow \infty } \sup \frac{G ( t )}{t}, \\& \lim_{t\rightarrow \infty } \sup \frac{\ln I(t)}{t} \\& \quad \leq \biggl( \frac{\alpha \varphi ( \mu + \sigma _{2} )}{\mu ( \mu + \sigma _{2} +\theta )} - \frac{1}{2} \beta _{3}^{2} - ( \mu +\delta +\tau ) \biggr) < 0 \\& \quad = ( \mu +\delta +\tau ) \biggl( \frac{\alpha \varphi ( \mu + \sigma _{2} )}{\mu ( \mu + \sigma _{2} +\theta ) ( \mu +\delta +\tau )} - \frac{1}{2 ( \mu +\delta +\tau )} \beta _{3}^{2} -1 \biggr) < 0 \\& \quad = ( \mu +\delta +\tau ) \bigl( R_{0}^{S} -1 \bigr). \end{aligned}$$

Obviously, $( \mu +\delta +\tau ) >0$, and therefore

3.30 $$\begin{aligned}& R_{0}^{S} -1< 0 \\& \quad \Rightarrow\quad R_{0}^{S} < 1. \end{aligned}$$

Therefore our disease free-equilibrium point is locally asymptotically stable if and only if

$$ R_{0}^{S} < 1. $$

 □

### Global stability of disease-free equilibrium point

#### Theorem 3.4

*If* $R_{0}^{D} <1$, *then* $E_{0}$ *is globally asymptotically stable in* Ω.

#### Proof

Consider a Lyaponuv function

3.31 $$ L ( t ) = \biggl[ \varphi +\alpha \delta + \frac{\varphi \theta }{ ( \mu +\delta )} \biggr] I ( t ). $$

Then differentiating Eq. (3.31), we get

3.32 $$\begin{aligned} \frac{dL}{dt}& = \biggl[ \varphi +\alpha \delta + \frac{\varphi \theta }{ ( \mu +\delta )} \biggr] \frac{dI}{dt} \\ &= \biggl[ \varphi +\alpha \delta + \frac{\varphi \theta }{ ( \mu +\delta )} \biggr] \bigl( \alpha S ( t ) I ( t ) -(\mu +\delta +\tau )I(t) \bigr) \\ &= \biggl[ \varphi +\alpha \delta + \frac{\varphi \theta }{ ( \mu +\delta )} \biggr] \bigl( \alpha S ( t ) - ( \mu +\delta +\tau ) \bigr) I ( t ) \\ &= \biggl[ \varphi +\alpha \delta + \frac{\varphi \theta }{ ( \mu +\delta )} \biggr] ( \mu +\delta + \tau ) \biggl( \frac{\alpha S ( t )}{ ( \mu +\delta +\tau )} -1 \biggr) I ( t ). \end{aligned}$$

Solving Eq. (3.32) at $S_{0} = \frac{\varphi (\mu + \sigma _{2} )}{\mu (\mu + \sigma _{2} +\theta )}$, we get

3.33 $$\begin{aligned} \frac{dL}{dt} &= \biggl[ \varphi +\alpha \delta + \frac{\varphi \theta }{ ( \mu +\delta )} \biggr] ( \mu +\delta +\tau ) \biggl( \frac{\alpha \varphi (\mu + \sigma _{2}}{\mu (\mu + \sigma _{2} +\theta ) ( \mu +\delta +\tau )} -1 \biggr) I ( t ) \\ &= \biggl[ \varphi +\alpha \delta + \frac{\varphi \theta }{ ( \mu +\delta )} \biggr] ( \mu +\delta + \tau ) \bigl( R_{0}^{D} -1 \bigr) I ( t ). \end{aligned}$$

Clearly, $[ \varphi +\alpha \delta + \frac{\varphi \theta }{ ( \mu +\delta )} ] ( \mu +\delta +\tau ) >0$. Then for $\frac{dL}{dt} \leq 0$, we must have $R_{0}^{D} -1<0$.

Thus every solution of equation system (2.1) with initial conditions in the domain Ω approaches disease-free equilibrium point $E_{0}$ as *t* goes to infinity whenever $R_{0}^{D} <1$.

Hence $E_{0}$ is globally asymptotically stable in the domain Ω. □

### Endemic equilibrium point

An endemic equilibrium point is a point in which the disease persists in the population, and we denote it as $E_{1} = S^{*}, V^{*}, I^{*}, T^{*}, R^{*}$ different from zero. Now $E_{1}$ can be obtained by equating all system of model Eq. (2.1) to zero:

3.34 $$ \frac{dS}{dt} = \frac{dV}{dt} = \frac{dI}{dt} = \frac{dT}{dt} = \frac{dR}{dt} =0. $$

Then after certain steps, we get

$$\begin{aligned}& S^{*} = \frac{ ( \varphi ( \mu + \rho +\omega ) + \sigma _{1} \rho \delta ) ( \mu +\theta )}{\alpha ( \mu + \rho +\omega )}, \\& V^{*} = \frac{\theta ( ( \varphi ( \mu + \rho +\omega ) + \sigma _{1} \rho \delta ) ( \mu +\theta ) )}{\alpha ( \mu + \rho +\omega ) ( \mu + \sigma _{2} )}, \\& I^{*} = \frac{ ( \mu + \rho +\omega )}{ ( \varphi ( \mu + \rho +\omega ) + \sigma _{1} \rho \delta ) ( \mu +\theta ) - ( \mu + \rho +\omega ) ( \mu +\delta +\tau )}, \\& T^{*} = \frac{\delta }{ ( \varphi ( \mu + \rho +\omega ) + \sigma _{1} \rho \delta ) ( \mu +\theta ) - ( \mu + \rho +\omega ) ( \mu +\delta +\tau )}, \\& R^{*} = \frac{\rho \delta }{(\mu + \sigma _{1} ) ( \varphi ( \mu + \rho +\omega ) + \sigma _{1} \rho \delta ) ( \mu +\theta ) - ( \mu + \rho +\omega ) ( \mu +\delta +\tau )}. \end{aligned}$$

Therefore the endemic equilibrium point of equation system (2.1) is

$$ E_{1} = \begin{pmatrix} \frac{ ( \varphi ( \mu + \rho +\omega ) + \sigma _{1} \rho \delta ) ( \mu +\theta )}{\alpha ( \mu + \rho +\omega )}, \frac{\theta ( ( \varphi ( \mu + \rho +\omega ) + \sigma _{1} \rho \delta ) ( \mu +\theta ) )}{\alpha ( \mu + \rho +\omega ) ( \mu + \sigma _{2} )} \\ \frac{ ( \mu + \rho +\omega )}{ ( \varphi ( \mu + \rho +\omega ) + \sigma _{1} \rho \delta ) ( \mu +\theta ) - ( \mu + \rho +\omega ) ( \mu +\delta +\tau )} \\ \frac{\delta }{ ( \varphi ( \mu + \rho +\omega ) + \sigma _{1} \rho \delta ) ( \mu +\theta ) - ( \mu + \rho +\omega ) ( \mu +\delta +\tau )} \\ \frac{\rho \delta }{(\mu + \sigma _{1} ) ( \varphi ( \mu + \rho +\omega ) + \sigma _{1} \rho \delta ) ( \mu +\theta ) - ( \mu + \rho +\omega ) ( \mu +\delta +\tau )} \end{pmatrix}. $$

### Stability analysis of endemic equilibrium point

In this section, we study the stability of model (2.1) at the endemic equilibrium point.

#### Theorem 3.5

*When* $R_{0}^{D} >1$, *the endemic equilibrium point* ($E_{1}$) *of equation system* (2.1) *is locally asymptotically stable at* $E_{1} = \{ S^{*}, V^{*}, I^{*}, T^{*}, R^{*} \} $.

#### Proof

Consider the Jacobian matrix at the endemic equilibrium point:

3.35 $$ J_{( E_{1} )} = \begin{pmatrix} -(\alpha I^{*} +\mu +\theta ) & \sigma _{2} & -\alpha S^{*} & 0 & \sigma _{1}\\ \theta & -(\mu + \sigma _{2} ) & 0 & 0 & 0\\ \alpha I^{*} & 0 & \alpha S^{*} -(\mu +\delta +\tau ) & 0 & 0\\ 0 & 0 & \delta & -(\mu +\rho +\omega ) & 0\\ 0 & 0 & 0 & \rho & -(\mu + \sigma _{2} ) \end{pmatrix}. $$

The characteristic polynomial equation of $J_{( E_{1} )}$ is

3.36 $$ P(\lambda ) = \lambda ^{5} + M_{1} \lambda ^{4} + M_{2} \lambda ^{3} + M_{3} \lambda ^{2} + M_{4} \lambda + M_{5}, $$

where

$$\begin{aligned}& M_{1} = K_{3} + K_{4} + K_{5} + K_{3} ( K_{4} + K_{5} ) + K_{4} K_{5} +\theta \sigma _{2}, \\& M_{2} = (K_{3} + K_{4} + K_{5} ) ( 1+ K_{1} + K_{2} ) + K_{1} K_{2}, \\& M_{3} = K_{3} K_{4} K_{5} + K_{3} \theta \sigma _{2} + ( K_{1} + K_{2} ) \bigl( K_{3} ( K_{4} + K_{5} ) + K_{4} K_{5} +\theta \sigma _{2} \bigr) + K_{1} K_{2} (K_{3} + K_{4} + K_{5} ), \\& M_{4} = (K_{1} + K_{2} ) ( K_{3} K_{4} K_{5} + K_{3} \theta \sigma _{2} ) + K_{1} K_{2} \bigl( K_{3} ( K_{4} + K_{5} ) + K_{4} K_{5} +\theta \sigma _{2} \bigr), \\& M_{5} = (K_{1} K_{2} (K_{3} K_{4} K_{5} + K_{3} \theta \sigma _{2} )- \sigma _{1} \delta \rho K_{5} \end{aligned}$$

with

$$\begin{aligned}& K_{1} = ( \mu + \sigma _{1} ), \\& K_{2} = ( \mu +\rho +\omega ), \\& K_{3} =\alpha S^{*} - ( \mu +\delta +\tau ), \\& K_{4} = \bigl(\alpha I^{*} +\mu +\theta \bigr), \\& K_{5} = ( \mu + \sigma _{2} ). \end{aligned}$$

Hence by the Routh-Hurwitz stability criterion the endemic equilibrium point is locally asymptotically stable if

$$\begin{aligned}& M_{1} >0,\qquad M_{2} >0,\qquad M_{3} >0,\qquad M_{4} >0,\qquad M_{5} >0, \\& M_{1} M_{2} M_{3} > M_{3}^{2} + M_{1}^{2} M_{4}, \\& M_{1} M_{4} - M_{5} \bigl( M_{1} M_{2} M_{3} - M_{3}^{2} - M_{1}^{2} M_{4} \bigr) > M_{5} ( M_{1} M_{2} - M_{3} )^{2} + M_{1} M_{5}^{2}. \end{aligned}$$

This completes the proof. □

#### Theorem 3.6

*When* $R_{0}^{D} >1$, *the endemic equilibrium* $E_{1}$ *is globally asymptotically stable*.

#### Proof

To show the global asymptotic stability at endemic equilibrium point, we define the following Lyapunov function:

3.37 $$ \begin{aligned}[b] L \bigl( S^{*}, V^{*}, I^{*}, T^{*}, R^{*} \bigr) &= \biggl( S- S^{*} - S^{*} \ln \frac{S^{*}}{S} \biggr) + \biggl( V- V^{*} - V^{*} \ln \frac{V^{*}}{V} \biggr)\\ &\quad {} + \biggl( I- I^{*} - I^{*} \ln \frac{I^{*}}{I} \biggr) + \biggl( T- T^{*} - T^{*} \ln \frac{T^{*}}{T} \biggr)\\ &\quad {} + \biggl( R- R^{*} - R^{*} \ln \frac{R^{*}}{R} \biggr). \end{aligned} $$

By calculating the derivatives of $L ( S^{*}, V^{*}, I^{*}, T^{*}, R^{*} )$ with respect to *t* we get

3.38 $$\begin{aligned}& \begin{aligned}[b] \frac{dL}{dt} &= \biggl( \frac{S- S^{*}}{S} \biggr) \frac{dS}{dt} + \biggl( \frac{V- V^{*}}{V} \biggr) \frac{dV}{dt} + \biggl( \frac{I- I^{*}}{I} \biggr) \frac{dI}{dI} \\ &\quad {}+ \biggl( \frac{T- T^{*}}{T} \biggr) \frac{dT}{dt} + \biggl( \frac{R- R^{*}}{R} \biggr) \frac{dR}{dt}, \end{aligned} \end{aligned}$$

3.39 $$\begin{aligned}& \begin{aligned}[b] \frac{dL}{dt} &= \biggl( \frac{S- S^{*}}{S} \biggr) \bigl( \varphi + \sigma _{1} R+ \sigma _{2} V- \bigl( \alpha I ( t ) +\theta +\mu \bigr) S \bigr) \\ &\quad {}+ \biggl( \frac{V- V^{*}}{V} \biggr) \bigl( \theta S- ( \mu + \sigma _{2} ) V \bigr)+ \biggl( \frac{I- I^{*}}{I} \biggr) \bigl( \alpha IS- ( \mu +\delta +\tau ) I \bigr) \\ &\quad {}+ \biggl( \frac{T- T^{*}}{T} \biggr) \bigl( \delta I- ( \mu +\rho +\omega ) T \bigr)+ \biggl( \frac{R- R^{*}}{R} \biggr) \bigl( \rho T- ( \mu + \sigma _{1} ) R \bigr). \end{aligned} \end{aligned}$$

Then simplifying Eq. (3.39), we have

3.40 $$\begin{aligned}& \begin{aligned}[b] \frac{dL}{dt} &=\varphi -\mu S- \sigma _{1} R \frac{S^{*}}{S} - \sigma _{2} V \frac{S^{*}}{S} + ( \alpha I+\theta +\mu ) S^{*} -\mu V-\theta S \frac{V^{*}}{V}\\ &\quad {} + ( \mu + \sigma _{2} ) V^{*} - ( \mu +\tau ) I -\alpha S I^{*} + ( \mu +\delta +\tau ) I^{*} - ( \mu +\omega ) T-\alpha I \frac{T^{*}}{T} \\ &\quad {}+ ( \mu +\rho +\omega ) T^{*} -\mu R-\rho T \frac{R^{*}}{R} + ( \mu + \sigma _{1} ) R^{*}, \end{aligned} \end{aligned}$$

3.41 $$\begin{aligned}& \begin{aligned}[b] \frac{dL}{dt} &= \bigl( ( \varphi +\alpha I+\theta +\mu ) S^{*} + ( \mu + \sigma _{2} ) V^{*} + ( \mu +\delta +\tau ) I^{*} + ( \mu +\rho +\omega ) T^{*} + ( \mu + \sigma _{1} ) R^{*} \bigr) \\ &\quad {}- \biggl( \mu S+ \sigma _{1} R \frac{S^{*}}{S} + \sigma _{2} V \frac{S^{*}}{S} +\mu V+\theta S \frac{V^{*}}{V} + ( \mu + \tau ) I+\alpha S I^{*} \\ &\quad {}+ ( \mu +\omega ) T+\alpha I \frac{T^{*}}{T} +\mu R+\rho T \frac{R^{*}}{R} \biggr). \end{aligned} \end{aligned}$$

Now let us take the parameters of positive coefficients as *U* and the negative ones as *V*. Then Eq. (3.41) becomes

$$ \frac{dL}{dt} =U-V. $$

For $U < V$, we have $S = S^{*}$, $V = V^{*}$, $I = I^{*}$, $T = T^{*}$, $R = R^{*}$ if and only if $\frac{dL}{dt} < 0$ and also $\frac{dL}{dt} = 0 $. The largest solution of compact set in $E_{1} \{( S^{*}, V^{*}, I^{*}, T^{*}, R^{*} ) \in \Omega : \frac{dL}{dt} = 0\}$ is the singleton of $E_{1}$.

Therefore by Lasalle’s invariant principle this indicates that the endemic equilibrium $E_{1}$ is globally asymptotically stable if $U < V$. □

### Sensitivity analysis and its interpretations

We determine the sensitivity of each parameter on the basic reproduction number of the model. We apply the following sensitivity index formula:

$$ P_{m_{i}}^{R_{0}} = \frac{\partial R_{0}}{\partial m_{i}} x \frac{m_{i}}{R_{0}}, $$

where $m_{i}$ are the parameters of the basic reproduction number. Here

$$\begin{aligned}& P_{\varphi }^{R_{0}} = \frac{\partial R_{0}}{\partial \varphi } x \frac{\varphi }{R_{0}} =1>0, \\& P_{\alpha }^{R_{0}} = \frac{\partial R_{0}}{\partial \alpha } x \frac{\alpha }{R_{0}} =1>0, \\& P_{\sigma _{2}}^{R_{0}} = \frac{\partial R_{0}}{\partial \sigma _{2}} x \frac{\sigma _{2}}{R_{0}} = \frac{\alpha \varphi \theta }{(\mu + \sigma _{2} +\theta )} >0, \\& P_{\delta }^{R_{0}} = \frac{\partial R_{0}}{\partial \delta } x \frac{\delta }{R_{0}} =- \frac{\alpha \varphi ( \mu + \sigma _{2} )}{ ( \mu +\delta \tau )} < 0, \\& P_{\theta }^{R_{0}} = \frac{\partial R_{0}}{\partial \theta } x \frac{\theta }{R_{0}} =- \frac{\alpha \varphi ( \mu + \sigma _{2} )}{ ( \mu + \sigma _{2} +\theta )} < 0, \\& P_{\tau }^{R_{0}} = \frac{\partial R_{0}}{\partial \tau } x \frac{\tau }{R_{0}} =- \frac{\alpha \varphi ( \mu + \sigma _{2} )}{ ( \mu +\delta \tau )} < 0. \end{aligned}$$

The sensitivity analysis interpretation of our basic reproduction number is described as follows. The parameters that have negative sensitivity indices (*δ*, *τ*, *θ*) have the effect of reducing the burden of COVID-19 from the community if the values of the two parameters are decreasing (which means that the basic reproduction number of the disease decreases as their parameter values decrease). Also, those parameters with positive sensitivity indices ($\sigma _{2}$, *φ*, *α*) have an important role in the expansion of COVID-19 in the community if their values increase (this means that if their parameter values increase, then the secondary infection in the community increases). Therefore, so as to minimize the disease from the community, it is vital to decrease the positive indices and increase the negative indices. In the study of sensitivity, increasing the human mortality rate to control disease epidemic is not ethically acceptable, and hence we do not consider it. See Table 2 for more information.

**Table 2:** Sensitivity indices

Parameter symbol	Description	Sensitivity indices
*φ*	recruitment rate	+ve
*α*	contact rate	+ve
$\sigma _{2}$	lose rate of immunity by vaccination	+ve
*δ*	treatment rate	−ve
*τ*	death causing death rate	−ve
*θ*	vaccination rate	−ve

## Numerical simulation results and discussions

In this section, we obtain the numerical simulation results of the deterministic and stochastic models of COVID-19. To display the results, we used Maple 18 and MATLAB-software. Now the graphs listed below demonstrate the results using the parameter values and initial conditions of the developed model. Here we have taken the parameter and variable values by assumption, estimation, and from recently published papers; they are listed in Table 3. Moreover, $S ( 0 ) = 50$, $V ( 0 ) = 35$, $I ( 0 ) =20$, $T ( 0 ) = 15$, and $R(0) = 10$ are the initial values.

**Table 3:** Values of parameters, state variables, and their sources for numerical simulation

Parameters and variables	Values	Sources
*φ*	0.008	[23]
*α*	0.0143	[19]
*ρ*	0.0012	[23]
*μ*	0.016	[19]
*δ*	0.004	assumed
*θ*	0.01	estimated
$\sigma _{1}$	0.15	[19]
$\sigma _{2}$	0.0005	estimated
*ω*	0.002	assumed
*τ*	0.017	estimated

### Deterministic and stochastic trends of the model

**Figure 2:**
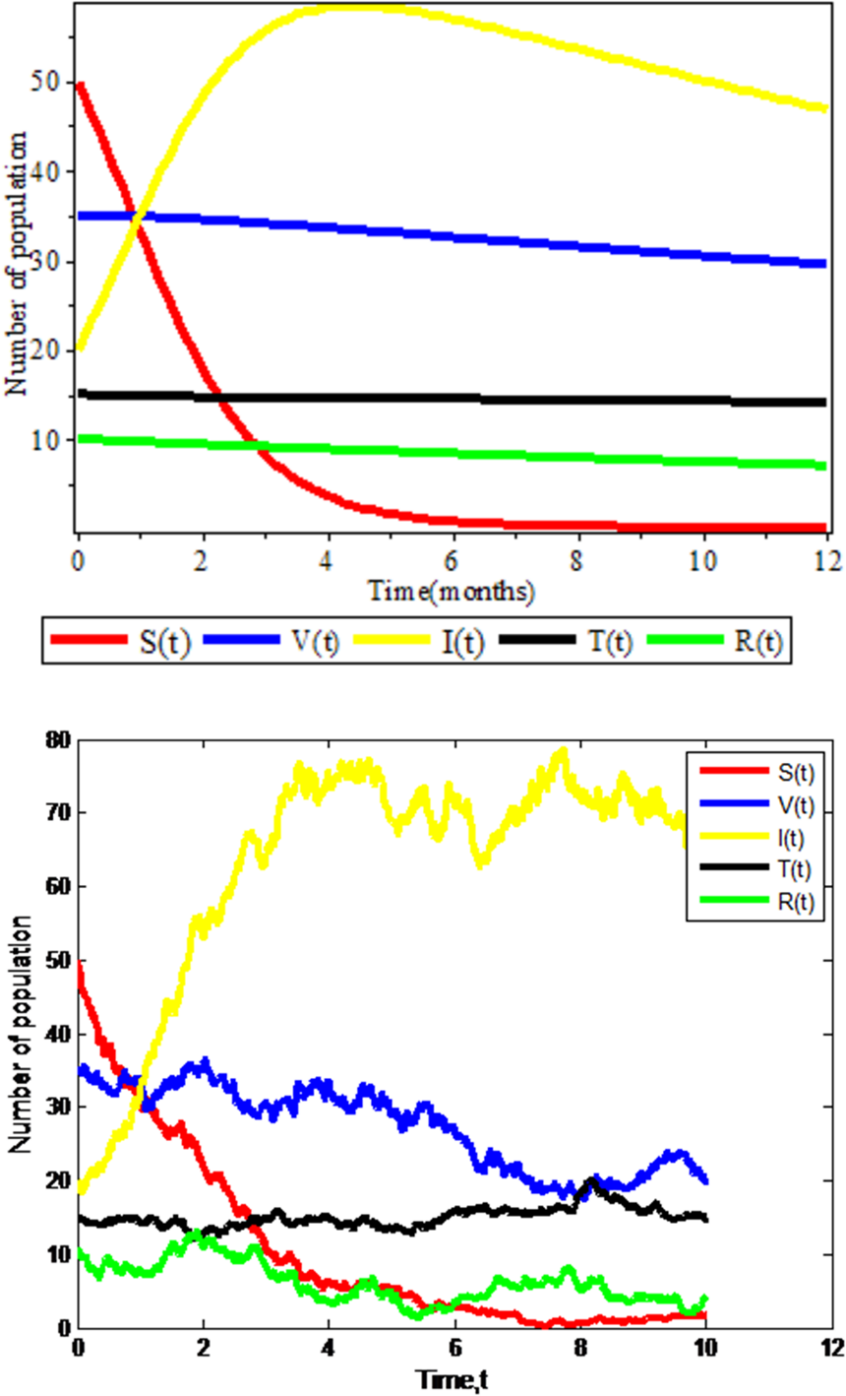
Graphs of deterministic and stochastic Covid-19 models

From the parameter values in Table 3 we obtained our basic reproduction numbers $R_{0}^{D} =0.0195522$ and $R_{0}^{S} = -1.19667$. We see that $R_{0}^{D} <1$ and $R_{0}^{S} <1$, which means that only susceptible population and vaccinated individuals are present and that infected, treated, and recovered populations are reduced to zero. This indicates that the model is asymptotically stable at $R_{0} <1$ for both deterministic and stochastic approaches, and this satisfies our theorem. This is verified numerically in Fig. 2.

Figure 2 is plotted using combination of all compartments and by taking fixed values of all parameters. From Fig. 2 we observe that in both deterministic and stochastic approaches the number of infectious population will increase and susceptible population will decrease after a long period of time when the susceptible individuals contact with infected individuals. In addition, in the deterministic case the graph shows a smooth curve, and in the stochastic case, we see the zigzagging properties (which imply that there is a probability for susceptible individuals to be infected by COVID-19 when infected individuals contact with susceptible one). Therefore from this we can conclude that the stochastic approach is more advisable for such kinds of disease.

### Effects of contact rate on infected individuals

**Figure 3:**
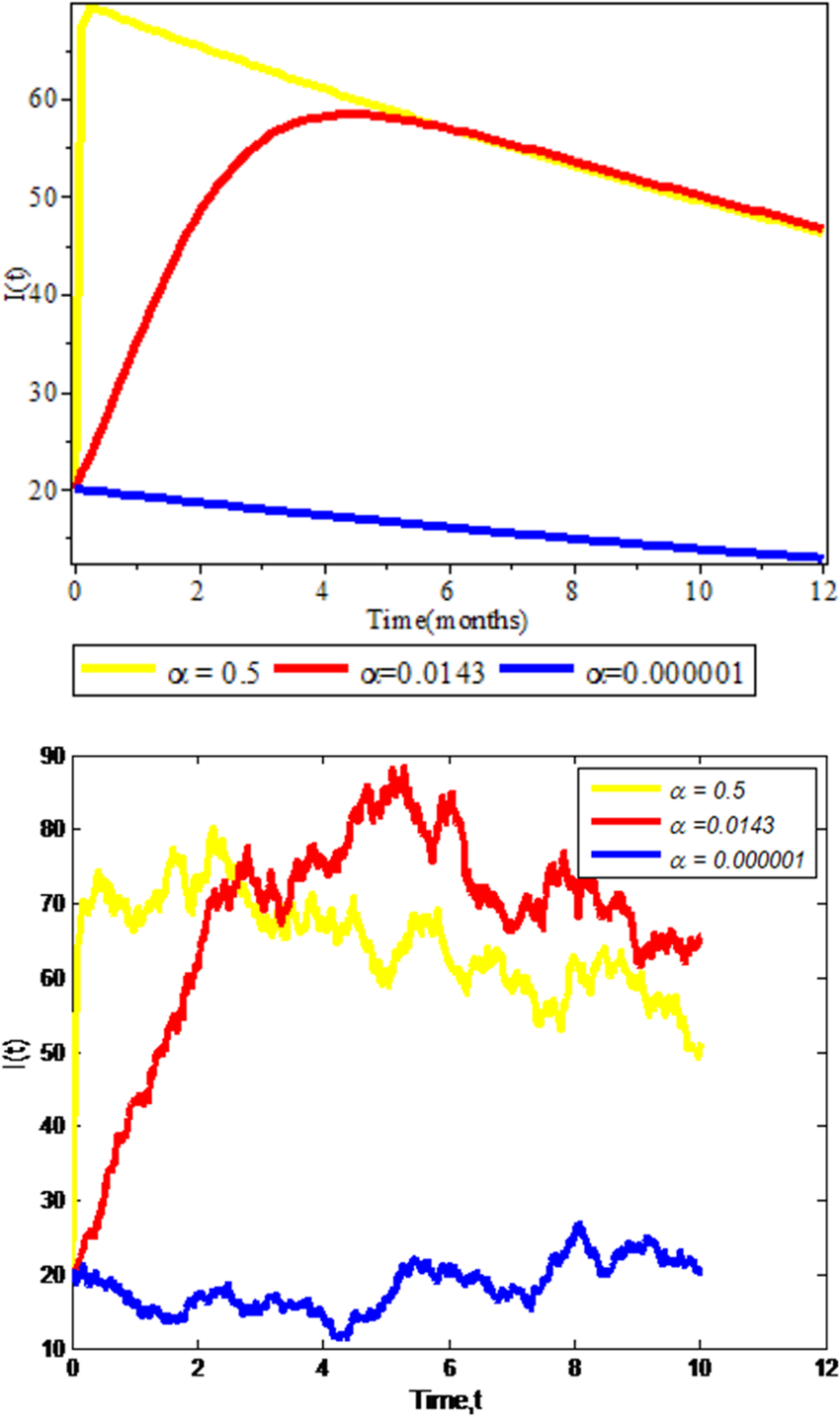
Effect of contact rate on COVID-19 expansion

As shown in Fig. 3, the simulation results of the effect of contact rate on infectious individuals $I(t)$ are illustrated. This figure is displayed by varying the values of contact rate *α* and keeping the remaining parameter values unchanged. When the values of contact rate is large ($\alpha = 0.5$), there is high possibility for the population to be infected by COVID-19, and for small values ($\alpha = 0.000001$), there is low probability for the individual to be infected by COVID-19. Moreover, the amount of infectious individuals increases as the contact rate increases in both deterministic and stochastic approaches. Therefore we strongly advise for the concerned body that by decreasing contact with COVID-19 infected individuals it is possible to reduce the disease in the community.

### Effects of treatment rate on infected individuals

**Figure 4:**
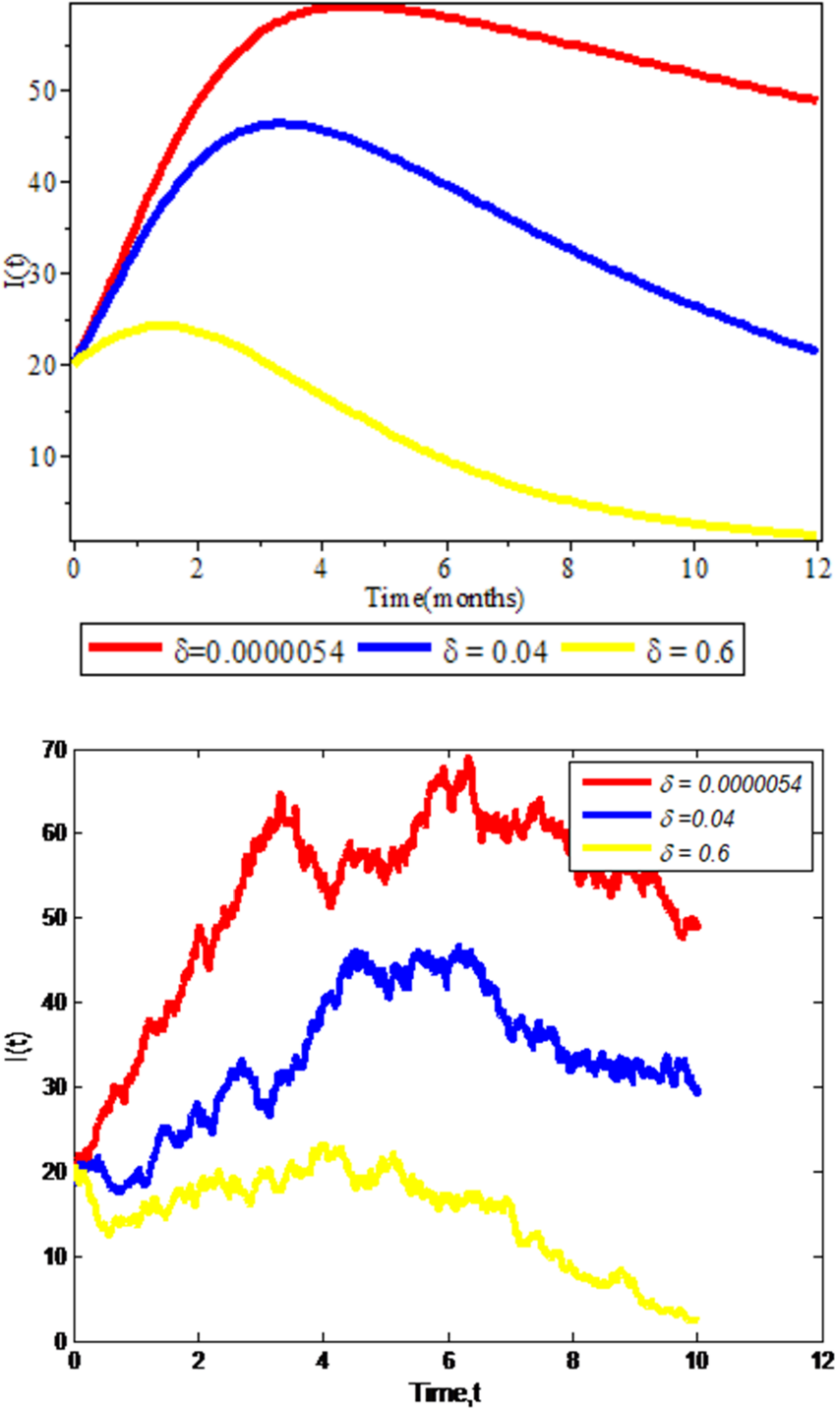
Effect of treatment rate on Covid-19 expansion

We investigate the experimental results of impacts of treatment rate on infectious population $I(t)$. As we see in Fig. 4, running numerical simulation results for deterministic approach is faster than for the stochastic approach, which clearly implies that the deterministic approach does not consider any probability (random properties) as the stochastic approach does. Besides, the infectious individuals become reduced by increasing treatment rate *δ* in both approaches. From this we can conclude that by treating the infectious individuals the infected population goes to treated one and COVID-19 will be eliminated from the community.

### Effects of recovery rate on recovered individuals

**Figure 5:**
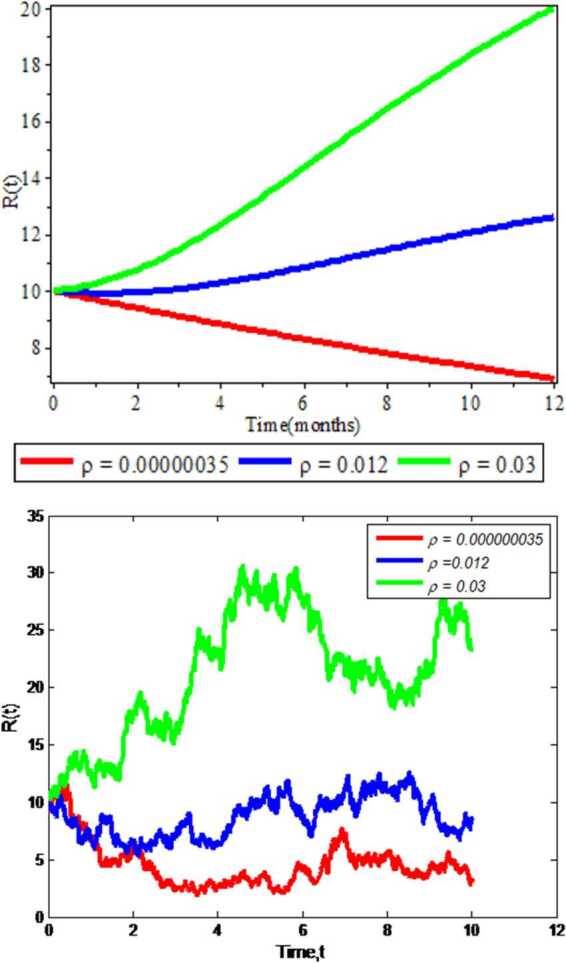
Effect of recovery rate on recovered individuals

In this subsection, we get the numerical simulation results of recovery rate *δ* on the amount of recovered individuals $R(t)$. In Fig. 5 we display the simulation results by keeping different values of recovery rate ($\rho = 0.00000035$, $\rho =0.012$, $\rho = 0.03$) and constant values of the other parameters. The figure clearly indicates that in the deterministic and stochastic approaches the increments of recovered individuals are obtained as the recovery rate in the individuals increase. Hence the greater the recovery rates, the more the individuals recover from COVID-19 in the community.

## Discussions and conclusions

In this investigation, we presented both deterministic and stochastic models for a novel coronavirus COVID-19 disease dynamics. Since COVID-19 by nature has a rapid transmission and variation of spreading ratio in different environments, in this study the stochastic approach performed better and provided more accurate results. This study is devoted to implement coronavirus mathematical model by containing vaccination class. Vaccination is the best pharmaceutical controlling strategy for COVID-19 disease. The basic reproduction number is calculated for both deterministic and stochastic approaches by using the next generation matrix method. We analyzed the existence and stability of a disease-free equilibrium point. The disease-free equilibrium points are locally asymptotically stable when $R_{0}^{D} <1$ and $R_{0}^{S} <1$. Simulation results and analysis of the model are performed using combinations of all compartments by varying the contact and treatment rates of infected individuals and the recovery rate on recovered individuals.

From our numerical results we found that in both deterministic and stochastic approaches when susceptible population contacts with infected individuals, there is high probability to increase the number of infectious population and to decrease the number of uninfected (susceptible) population. Moreover, increasing the contact rate on population has an impact on the rate of spread of COVID-19 in the community. This means that when the values of contact rate *α* are large enough, there is high probability for the individuals to be infected by novel coronavirus COVID-19. In addition to this, infectious individuals are reduced by increasing the treatment rate *δ*. Hence vaccination strategies are regarded as the most effective measures to prevent and control rapid transmission of novel coronavirus COVID-19 in the community. Thus every citizen can take vaccine to minimize the spread of the disease. Therefore we strongly recommend other potential researchers to study different vaccination phases, like first and second doses of vaccinations ($V_{1}$ and $V_{2}$), and extensions by using optimal control strategies to modify our model.

## Data Availability

The data we used for this research are from respective published and cited papers.
